# Draft Genome Sequence of *Raoultella terrigena* R1Gly, a Diazotrophic Endophyte

**DOI:** 10.1128/genomeA.00607-15

**Published:** 2015-06-11

**Authors:** M. Schicklberger, N. Shapiro, D. Loqué, T. Woyke, R. Chakraborty

**Affiliations:** a Lawrence Berkeley National Laboratory (LBNL), Berkeley, California, USA; b DOE Joint Genome Institute (JGI), Walnut Creek, California, USA; c LBNL DOE Joint BioEnergy Institute (JBEI), Emeryville, California, USA

## Abstract

*Raoultella terrigena* R1Gly is a diazotrophic endophyte isolated from surface-sterilized roots of *Nicotiana tabacum*. The whole-genome sequence was obtained to investigate the endophytic characteristics of this organism at the genetic level, as well as to compare this strain with its close relatives. To our knowledge, this is the first genome obtained from the *Raoultella terrigena* species and only the third genome from the *Raoultella* genus, after *Raoultella ornitholytic* and *Raoultella planticola*. This genome will provide a foundation for further comparative genomic, metagenomic, and functional studies of this genus.

## GENOME ANNOUNCEMENT

*Raoultella*, a genus named after the French bacteriologist Didier Raoult, comprises Gram-negative, oxidase-negative, facultative anaerobic organisms, with both respiratory and fermentative types of metabolism. *Raoultella* belongs to the family *Enterobacteriaceae*, and based on phylogenetic analysis of the 16S rRNA and *rpoB* genes, *Raoultella* was divided from the genus *Klebsiella* (1). We isolated *Raoultella* strain R1Gly after enriching surface-sterilized root samples of *Nicotiana tabacum* in nitrogen-free HGB medium (2), using glycerol as the sole carbon source. This strain grew optimally at 30°C, and its ability to fix atmospheric nitrogen (N) was confirmed by the presence of the *nifH* gene and by the acetylene reduction assay (3). The draft genome sequence revealed a 5.7-Mb genome with 57.84 mol% G+C content, which is comparable to those of the diazotrophs *Raoultella planticola* (5.8 Mb; 55.4 mol%) and *Raoultella ornithinolytica* S12 (5.5 Mb; 57.47 mol%) as well as the nondiazotrophs *Raoultella ornithinolytica* B6 (5.3 Mb; 55.75 mol%) and *Raoultella ornithinolytica* TNT (5.6 Mb; 55.5 mol%) (4–7). The COG predictions categorize 2,062 of the 5,239 protein-encoding genes as pertaining to metabolism and transport, 1,752 to intracellular processes, and 475 to extracellular processes and 229 as having unknown functions.

Interestingly, apart from genes for biological nitrogen fixation, strain R1Gly contains all necessary genes for the tryptophan-dependent production of the plant hormone indole acetic acid (*ipdC*) and the production of 2,3-butanediol and acetoin volatiles previously shown to promote growth in *Arabidopsis* (8).

The genomic DNA was isolated using a cetyltrimethylammonium bromide (CTAB)-based extraction from cells grown overnight at 30°C on LB medium. The draft genome of *Raoultella terrigena* R1Gly was generated at the DOE Joint Genome Institute (JGI) using the Pacific Biosciences (PacBio) sequencing technology (9). A Pacbio SMRTbell library was constructed and sequenced on the PacBio RS platform, which generated 322,267 filtered subreads totaling 639.3 Mbp. All general aspects of library construction and sequencing performed at the JGI can be found at http://www.jgi.doe.gov. The raw reads were assembled using HGAP (version: 2.2.0.p1) (10). The final draft assembly contained 10 contigs in 10 scaffolds, totaling 5.7 Mbp in size. The input read coverage was 151.9×.

Genes were identified using Prodigal (11), followed by a round of manual curation using GenePRIMP (12). The predicted coding sequences (CDSs) were translated and used to search the National Center for Biotechnology Information (NCBI) nonredundant database and the UniProt, TIGRFam, Pfam, KEGG, COG, and InterPro databases. The tRNAScan-SE tool (13) was used to find tRNA genes, whereas rRNA genes were found by searches against models of the rRNA genes built from SILVA (14). Other noncoding RNAs such as the RNA components of the protein secretion complex and RNase P were identified by searching the genome for the corresponding Rfam profiles using INFERence of RNA ALignment (INFERNAL). Additional gene prediction analysis and manual functional annotation were performed within the Integrated Microbial Genomes (IMG) platform (15) developed by the JGI.

### Nucleotide sequence accession numbers. 

This whole-genome shotgun project has been deposited at DDBJ/EMBL/GenBank under project accession no. LANE00000000. The version described in this paper is the version LANE00000000.1.
